# Isolating SARS-CoV-2 Strains From Countries in the Same Meridian: Genome Evolutionary Analysis

**DOI:** 10.2196/25995

**Published:** 2021-01-22

**Authors:** Emilio Mastriani, Alexey V Rakov, Shu-Lin Liu

**Affiliations:** 1 Systemomics Center, College of Pharmacy, Genomics Research Center State-Province Key Laboratories of Biomedicine-Pharmaceutics of China Harbin Medical University Harbin China; 2 HMU-UCCSM Centre for Infection and Genomics Harbin Medical University Harbin China; 3 Somov Institute of Epidemiology and Microbiology Vladivostok Russian Federation; 4 Department of Microbiology, Immunology and Infectious Diseases University of Calgary Calgary, AB Canada

**Keywords:** SARS-CoV-2, evolutionary analysis, episodic selective pressure, virus evolution, codon mutation, binding probability, evolution, genome, genetics, COVID-19, virus, strain, codon, pressure, mutation, structure, prediction, protein

## Abstract

**Background:**

COVID-19, caused by the novel SARS-CoV-2, is considered the most threatening respiratory infection in the world, with over 40 million people infected and over 0.934 million related deaths reported worldwide. It is speculated that epidemiological and clinical features of COVID-19 may differ across countries or continents. Genomic comparison of 48,635 SARS-CoV-2 genomes has shown that the average number of mutations per sample was 7.23, and most SARS-CoV-2 strains belong to one of 3 clades characterized by geographic and genomic specificity: Europe, Asia, and North America.

**Objective:**

The aim of this study was to compare the genomes of SARS-CoV-2 strains isolated from Italy, Sweden, and Congo, that is, 3 different countries in the same meridian (longitude) but with different climate conditions, and from Brazil (as an outgroup country), to analyze similarities or differences in patterns of possible evolutionary pressure signatures in their genomes.

**Methods:**

We obtained data from the Global Initiative on Sharing All Influenza Data repository by sampling all genomes available on that date. Using HyPhy, we achieved the recombination analysis by genetic algorithm recombination detection method, trimming, removal of the stop codons, and phylogenetic tree and mixed effects model of evolution analyses. We also performed secondary structure prediction analysis for both sequences (mutated and wild-type) and “disorder” and “transmembrane” analyses of the protein. We analyzed both protein structures with an ab initio approach to predict their ontologies and 3D structures.

**Results:**

Evolutionary analysis revealed that codon 9628 is under episodic selective pressure for all SARS-CoV-2 strains isolated from the 4 countries, suggesting it is a key site for virus evolution. Codon 9628 encodes the P0DTD3 (Y14_SARS2) uncharacterized protein 14. Further investigation showed that the codon mutation was responsible for helical modification in the secondary structure. The codon was positioned in the more ordered region of the gene (41-59) and near to the area acting as the transmembrane (54-67), suggesting its involvement in the attachment phase of the virus. The predicted protein structures of both wild-type and mutated P0DTD3 confirmed the importance of the codon to define the protein structure. Moreover, ontological analysis of the protein emphasized that the mutation enhances the binding probability.

**Conclusions:**

Our results suggest that RNA secondary structure may be affected and, consequently, the protein product changes T (threonine) to G (glycine) in position 50 of the protein. This position is located close to the predicted transmembrane region. Mutation analysis revealed that the change from G (glycine) to D (aspartic acid) may confer a new function to the protein—binding activity, which in turn may be responsible for attaching the virus to human eukaryotic cells. These findings can help design in vitro experiments and possibly facilitate a vaccine design and successful antiviral strategies.

## Introduction

The ongoing COVID-19 pandemic caused by the novel SARS-CoV-2 is the most threatening respiratory infection worldwide and has affected almost every country in the world. As of December 30, 2020, over 81 million people were infected with COVID-19, and more than 1.7 million deaths were reported. Many health institutions are attempting to produce effective vaccines against this virus infection, and several are now in the final stages of development before their application to human populations [1,2].

The SARS-CoV-2 genome shares approximately 82% sequence identity with SARS-CoV and MERS-CoV (Middle East respiratory syndrome coronavirus) and more than 90% sequence identity for essential enzymes and structural proteins. This high level of sequence identity suggests a common pathogenesis mechanism and, thus, therapeutic targeting. SARS-CoV-2 contains 4 structural proteins, including spike (S), envelope (E), membrane (M), and nucleocapsid (N) proteins [3]. The structure and the genome of SARS-CoV-2 are being extensively studied, but the results seem to be controversial. For example, a recent study found that the 2 integral membrane proteins (ie, envelope and membrane proteins) tend to evolve slowly by accumulating nucleotide mutations on their corresponding genes, but genes encoding nucleocapsid, viral replicase and spike proteins, which are regarded as important targets for the development of vaccines and antiviral drugs, tend to evolve faster [4]. However, other studies have shown that potential drug targets of SARS-CoV-2 are highly conserved [3].

The genome of SARS-CoV-2 is comprised of a single-stranded positive-sense RNA. A newly sequenced genome of SARS-CoV-2 was submitted to the NCBI genome database (NC_045512.2). The genetic makeup of SARS-CoV-2 is composed of 13-15 (including 12 functional) open reading frames (ORFs) containing ~30,000 nucleotides. The genome contains 38% of GC content and 11 protein-coding genes, together expressing 12 proteins [3].

The genomic characterization of 95 SARS-CoV-2 genomes revealed the 2 most common mutations that might affect the severity and spread of SARS-CoV-2 [5]. Another study highlighted the crucial genomic features that are unique to SARS-CoV-2 and 2 other deadly coronaviruses, SARS-CoV and MERS-CoV. These unique features correlate with the high fatality rate due to infection with these coronaviruses as well as their ability to switch hosts from animals to humans [6]. As a result, it can be speculated that the epidemiological and clinical features of these viruses may differ across countries or continents.

Genomic comparison of 48,635 SARS-CoV-2 genomes has shown that the average number of mutations per sample was 7.23, and most SARS-CoV-2 strains belong to one of the following 3 clades characterized by geographic and genomic specificity: clade G (Europe), clade L (Asia), and G-derived clade (North America) [7]. These results suggest custom-designed antiviral strategies based on the molecular specificities of SARS-CoV-2 in patients from different geographies [7]. Previous studies have also differentiated the 3 variants according to the geographic location (East Asia, Europe, and America) [8]. A more recent genome-wide analysis revealed that the frequency of amino acid mutations was higher in the genome sequences of SARS-CoV-2 strains from Europe (43.07%), followed by strains from Asia (38.09%) and North America (29.64%). However, case fatality rates remained higher in the European temperate countries, such as Italy, Spain, Netherlands, France, England, and Belgium [9].

The aim of this study was to compare the set of SARS-CoV-2 genomes of viral strains isolated from representative countries in the same meridian (longitude), namely, Italy, Sweden, and Congo, which have different climate conditions, to reveal similarities or differences in the patterns of possible evolutionary pressure signatures in their genomes.

## Methods

### Sequence Data

We obtained data from the Global Initiative on Sharing All Influenza Data (GISaid) repository and sampled all genomes available therein to that date (May 5, 2020), including the files congo-gisaid_hcov-19_2020_05_05_09.fasta with 75 entries, italy-gisaid_hcov-19_2020_05_05_10.fasta with 69 entries, sweden-gisaid_hcov-19_2020_05_05_10.fasta with 104 entries, and also the outgroup file brazil_gisaid_hcov-19_2020_05_15_04.fasta with 92 entries. The reference genome with accession number NC_045512.2 was downloaded from the GenBank repository.

### Evolution Model Analysis

We used the SARS-CoV-2 Wuhan-Hu-1 genome (RefSeq Acc. No. NC_045512.2) as the reference sequence and the VIRULIGN version 1.0.1 application [10] to perform multiple sequence alignment, with AliView version 1.26 application for visualizing the results of the analyses [11]. HyPhy 2.5.8 (MP) was used to perform recombination analysis by the genetic algorithm recombination detection method and conduct trimming, stop codon removal, and phylogenetic tree and mixed effects model of evolution (MEME) analyses [12]. The MEME web site was used to read JSON output files and generate MEME images and tables.

### RNA Secondary Structure Prediction

We used the RNA_fold web server (part of the Vienna RNA Websuite) to predict secondary structures of both the wild-type and mutated sequences [13], and the Forna package [14] to build the graph diagrams.

### Protein Analysis

Protein disorder analysis was conducted using MFDp2 [15], NetSurfP-2.0 [16], and SPOT-Disorder2 [17] applications. Transmembrane analysis of the protein was calculated using the TMHMM server v.2.0, MemBrain webserver [18], ProtScale [19], and TMpred [20] (scores normalized for comparison) on the Expasy website [21].

### 3D Protein Structure Prediction and Ontologies

Both protein structures were determined with an ab initio approach by using the Robetta webserver [22], whereas DeeProtein capsule from OCEAN CODE [23] was used to predict ontologies of the predicted proteins. 3D images of protein structures and their ontologies were released using PyMOL 2.4.0 [24].

## Results

### Codon 9628 Evolved Under Episodic Positive Selection

Mixed evolutionary analysis based on the MEME algorithm was conducted on the SARS-CoV-2 data from Italy, Sweden, and Congo (countries from the same geographic meridian) and Brazil (included as an outgroup). The investigation revealed codon 9628 was under episodic positive selective pressure across the countries, as depicted in Table 1.

**Table 1:** Mixed effects model of evolution (MEME_ analysis results showing data obtained from the evolutionary analysis of SARS-CoV-2 from Brazil, Congo, Italy, and Sweden. The top 3 sites for every country are shown, sorted by *P* value.

Country (ID)/Site		Partition	α	β^−^	*p^−^*	β^+^	*p^+^*	LRT	*P* value	Branches under selection	Total branch length	MEME LogL	Fixed effects likelihood LogL
**Brazil (BR)**													
	9628^a^	1	0	0	0.96	10,000	0.04	16.37	<.001	2	0.65	-27.28	-20.62
	9928	1	0	0	0.82	10,000	0.18	11.12	<.001	4	2.71	-31.03	-28.53
	81	1	0	0	0.04	1032.18	0.96	6.95	.01	5	1.49	-40.77	-40.77
**Congo (CG)**													
	9628^a^	1	0	0	0.97	10,000	0.03	10.89	<.001	1	0.25	-18.18	-13.54
	2884	1	0	0	0.45	1273.45	0.55	3.51	.08	5	0.60	-42.49	-42.37
	6541	1	0	0	0.97	10,000	0.03	2.73	.12	1	0.27	-12.94	-11.92
**Italy (IT)**													
	15	1	0	0	0.96	10,000	0.04	10.21	<.001	1	0.73	-15.90	-12.57
	9628^a^	1	0	0	0.97	1,0000	0.03	11.24	<.001	1	0.45	-17.66	-12.95
	4	1	0	0	0.89	10,000	0.11	7.25	.01	0	1.83	-13.11	-10.43
**Sweden (SE)**													
	9628^a^	1	0	0	0.96	9613.52	0.04	16.03	<.001	2	0.51	-27.43	-21.10
	4409	1	0	0	0.97	4356.70	0.03	7.68	.01	1	0.16	-15.63	-12.33
	4732	1	0	0	0.95	10,000	0.05	3.85	.07	2	0.74	-19.66	-18.78

^a^Indicates site 9628.

**Figure 1:**
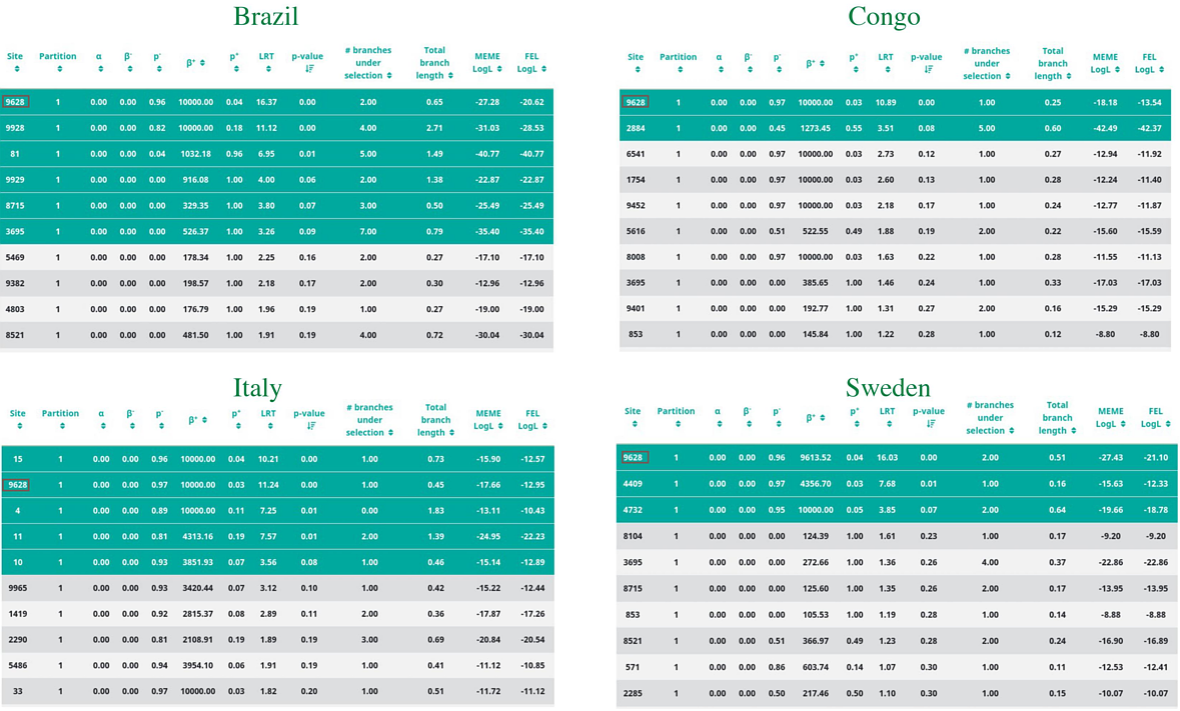
Mixed effects model of evolution site plot. Distribution of the *P* value over the sites in Brazil, Congo, Italy, and Sweden. The purple circle indicates site 9628 that was found to be under episodic selective pressure.

In this context, we use the term “site” as a synonym of codon, respecting the HyPhy terminology. The asymptotic *P* value was <.001 for episodic diversification at site 9628. Figure 1 shows the distribution of the *P* value across the sites for all 4 countries.

**Figure 2:**
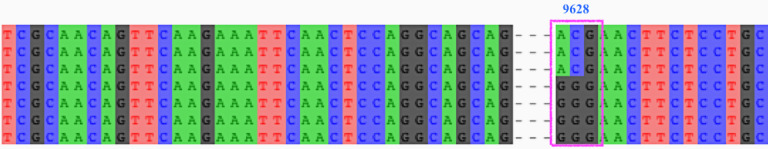
Part of the multiple sequence alignment from the Italian data showing the site 9628 under episodic selective pressure. The nucleotides mute from GGG to ACG.

A deep check of the multiple alignment data of the 4 countries revealed that the episodic positive selective pressure on site 9628 is a consistent mutation of the codon GGG to ACG, as shown in Figure 2.

### RNA Secondary Structure Prediction Changes

**Figure 3:**
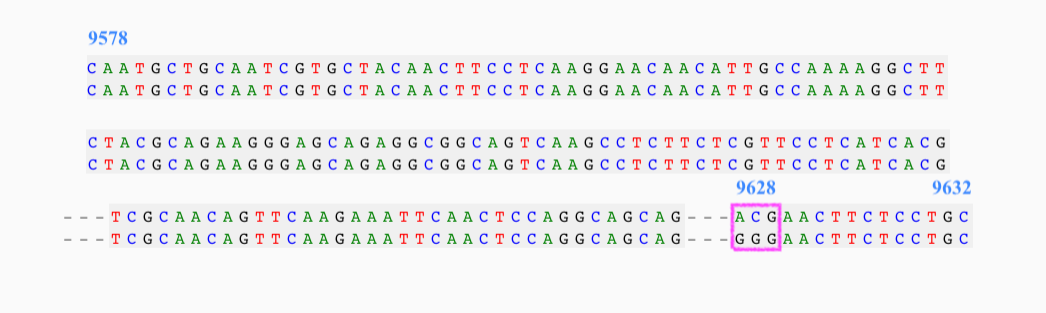
Nucleotide mutation over aligned sequences, illustrating the sequence considered to predict secondary structures in both mutated and wild-type proteins. Site position is indicated in blue, from the start codon (9578) to the open reading frame (9632).

**Figure 4:**
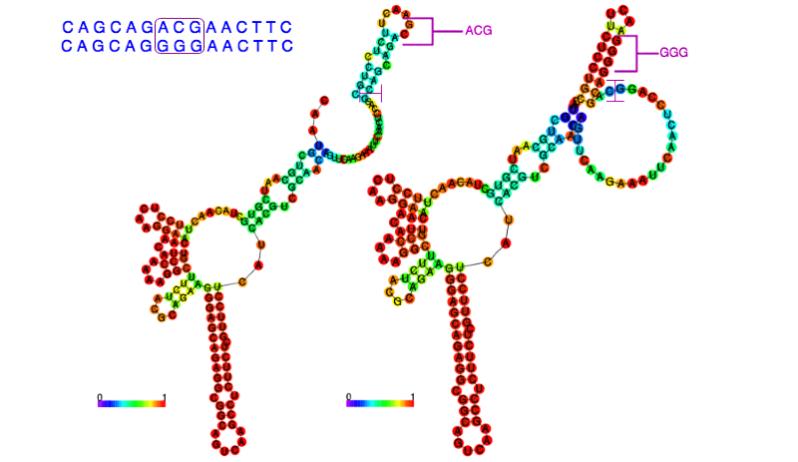
Secondary structure prediction. The 2 RNA diagrams exhibit structural modifications affected by the GGG to ACG mutation.

The prediction of secondary structure before and after mutation shows important differences, as shown by the mutation from GGG to ACG (Figure 3). The comparison between the 2 predicted secondary structures highlighted structural modifications at the top-right ring of the RNA conformation, as depicted in Figure 4, suggesting the GGG to ACG mutation was responsible for a significant modification of the RNA secondary structure.

### Protein Analysis

**Figure 5:**
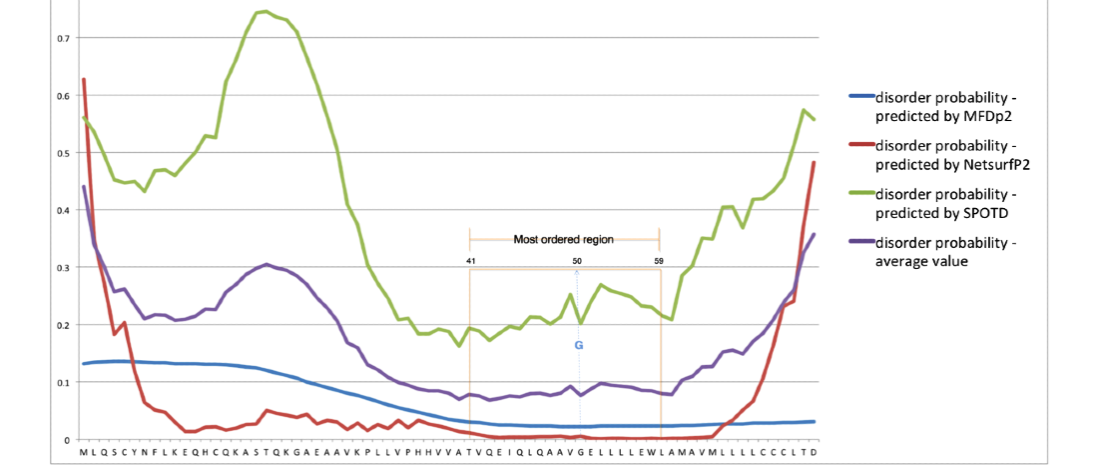
Disorder region analysis. The region 41-59 was found to have the lowest probability to be disordered. The orange lines delimit this region, and the blue dotted line outlines the position of G on the different curves.

**Figure 6:**
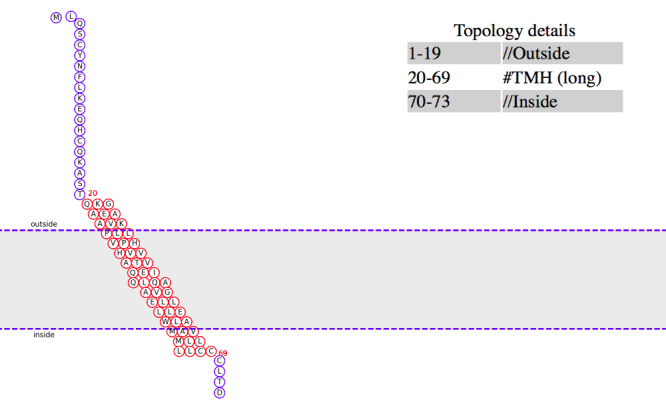
Topology diagram using the MemBrain v3. The illustration depicts the transmembrane topology of the sequence and highlights that the amino acid at position 50 (G) is positioned into the middle of the transmembrane region. Red: transmembrane helix (TMH); blue: loop.

**Figure 7:**
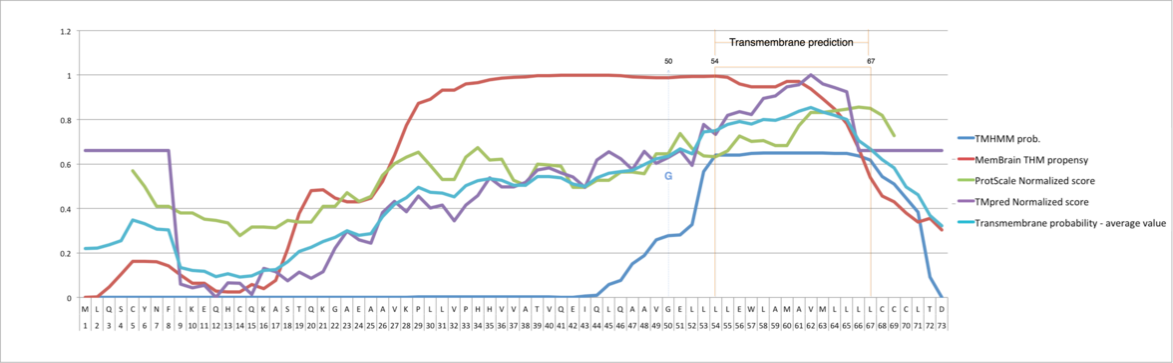
Transmembrane prediction. The region 54-67 was found to be the region with the highest probability to code for the transmembrane, and the G amino acid is near enough to suppose its involvement. The orange lines delimit this region, and the blue dotted line outlines the position of G on the different curves.

The analysis of the protein conducted for finding its disordered region turned out the positions from 41 to 59 to be more stable with the glycine (G) placed at the 50th position. We obtained results by using 3 different software tools and considering the average value for the probability of disorder, as shown in Figure 5 and reported in Table 2. Further analysis to locate the transmembrane region in the protein revealed that locations 54-67 were associated with this function. The analysis, conducted by using 4 distinct web applications and by evaluating the resultant average values, places the glycine (G) as near the transmembrane region to suppose its involvement. Table 3 reports the data showing the probabilities of each amino acid acting as the transmembrane. The transmembrane topology of the sequence (Figure 6) highlights the amino acid G at location 50 in the middle of the transmembrane region, and the distribution of the probabilities (Figure 7) corroborates this hypothesis.

**Table 2:** Protein disorder analysis results showing the probability of disorder for each position of the protein. The probabilities have been calculated using MFDp2, Netsurf, and SPOTD software.

Position	Amino acid sequence	Disorder probability values			
		MFDp2	NetsurfP2	SPOTD	Average value^a^
1	M	0.132	0.627823114	0.5607	0.440174371
2	L	0.134	0.347978383	0.5358	0.339259461
3	Q	0.135	0.270706475	0.4945	0.300068825
…					
39	T	0.03	0.010842944	0.1936	0.078147648
40	V	0.029	0.007660664	0.189	0.075220221
41	Q	0.027	0.004478907	0.172	0.067826302
42	E	0.025	0.00340931	0.1848	0.07106977
43	I	0.025	0.003887762	0.1968	0.075229254
44	Q	0.024	0.003997837	0.1927	0.073565946
45	L	0.023	0.00361518	0.2129	0.079838393
46	Q	0.023	0.004551574	0.2123	0.079950525
47	A	0.023	0.004939525	0.2011	0.076346508
48	A	0.022	0.005752307	0.2133	0.080350769
49	V	0.022	0.002826149	0.2524	0.092408716
50^b^	G	0.022	0.005828088	0.2013	0.076376029
51	E	0.022	0.001046103	0.24	0.087682034
52	L	0.023	0.000922468	0.2694	0.097774156
53	L	0.023	0.001263275	0.2588	0.094354425
54	L	0.023	0.001187441	0.2539	0.092695814
55	L	0.023	0.000650476	0.2483	0.090650159
56	E	0.023	0.000615434	0.2328	0.085471811
57	W	0.023	0.001080571	0.2302	0.08476019
58	L	0.023	0.000941573	0.2154	0.079780524
59	A	0.023	0.001573079	0.208	0.07752436
60	M	0.024	0.000997698	0.2853	0.103432566
61	A	0.024	0.00227783	0.3026	0.109625943
62	V	0.025	0.003362786	0.3503	0.126220929

^a^Average values of the disorder probability for each position.

^b^Amino acid G placed at position 50, inside the stable region.

**Table 3:** Transmembrane prediction results obtained using TMHMM, MemBrainTHM, ProtScale, and TMpred applications. Results from ProtScale and TMpred have been normalized for comparison with other probabilities.

Position	Amino acid sequence	TMHMM probability	MemBrain THM propensity	ProtScale normalized score	TMpred normalized score	Transmembrane probability, average value^a^
1	M	0	0.000191	N/A^b^	0.661425764	0.220538921
2	L	0	0.002851	N/A^b^	0.661425764	0.221425588
3	Q	0	0.046538	N/A^b^	0.661425764	0.235987921
…						
49	V	0.2594	0.987914	0.646	0.603358942	0.624168236
50^c^	G	0.27719	0.987914	0.646	0.629801679	0.63522642
51	E	0.28083	0.991702	0.736	0.660532428	0.667266107
52	L	0.32735	0.993857	0.67	0.594246918	0.646363479
53	L	0.56651	0.993857	0.637	0.778452743	0.743954936
54	L	0.63937	0.994522	0.632	0.73360729	0.749874822
55	L	0.64032	0.990459	0.659	0.818831517	0.777152629
56	E	0.64052	0.96027	0.726	0.835626228	0.790604057
57	W	0.64826	0.946819	0.701	0.822583527	0.779665632
58	L	0.6493	0.947424	0.706	0.895122387	0.799461597
59	A	0.64928	0.947424	0.683	0.905663748	0.796341937
60	M	0.64927	0.970735	0.683	0.947293193	0.812574548
61	A	0.64924	0.970735	0.773	0.955511881	0.83712172
62	V	0.64903	0.937507	0.831	1	0.85438425
63	M	0.64893	0.892506	0.831	0.960871896	0.833326974
64	L	0.6482	0.846403	0.84	0.942826514	0.819357379
65	L	0.64758	0.781733	0.847	0.924066464	0.800094866
66	L	0.63557	0.670387	0.856	0.661425764	0.705845691
67	L	0.61835	0.539353	0.851	0.661425764	0.667532191
68	C	0.5428	0.455615	0.819	0.661425764	0.619710191
69	C	0.51009	0.430385	0.728	0.661425764	0.582475191
70	C	0.44702	0.380525	N/A^b^	0.661425764	0.496323588

^a^Average values of the probability for each position.

^b^The window size used for the profile computation is 9, so the score is not applicable for positions 1-4 and 70-73.

^c^Amino acid G placed at position 50, inside the stable region.

### 3D Protein Analysis

**Figure 8:**
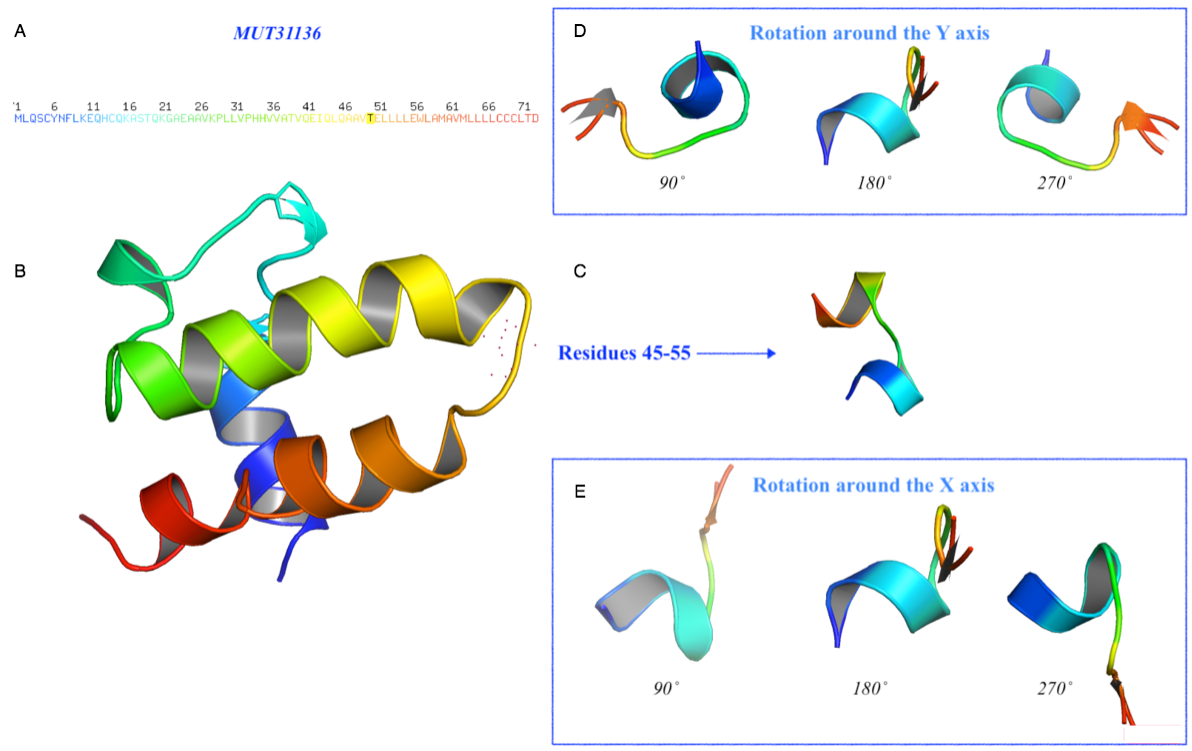
Prediction of the 3D structure for the mutated protein of SARS-CoV-2. The model MUT31136 represents the predicted 3D model of the protein subject to mutation. (A) Amino acid sequence colored by the spectrum range, with the mutated amino acid indicated in black color at position 50 (T). (B) The protein has been oriented to facilitate the comparison and residue 50 is represented with red dots. (C) Details of the residues 45-55 and their rotation (D) around the Y-axis and (E) around the X-axis with a step of 90˚.

**Figure 9:**
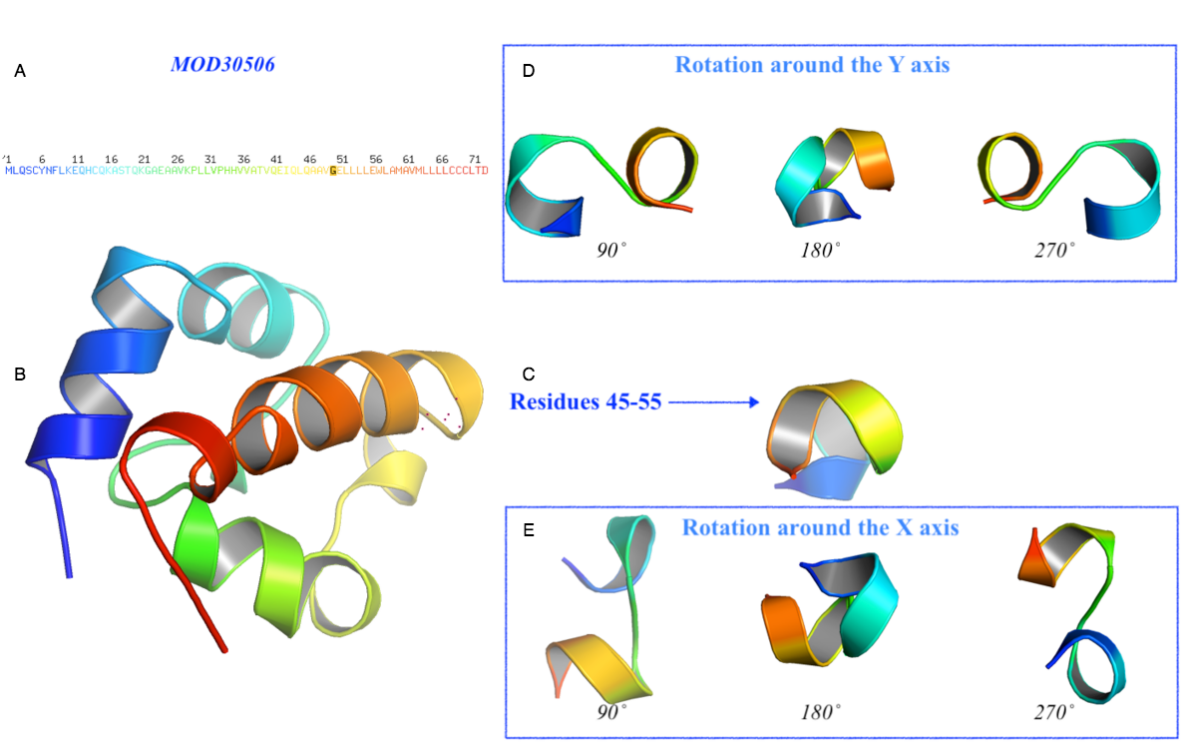
Prediction of the 3D structure of the unchanged protein. The model MOD30506 represents the predicted 3D model of the wild-type protein. (A) Amino acid sequence colored by the spectrum range, with the investigated amino acid indicated in black color at position 50 (G). (B) The protein has been oriented to facilitate the comparison and the residue 50 is represented with the red dots. (C) Details of the residues 45-55 and their rotation (D) around the Y-axis and (E) around the X-axis with a step of 90˚.

To characterize the deduced protein P0DTD3.1, we predicted the 3D structures for both the wild-type and mutated protein sequences using an ab initio approach. According to the preliminary clue from the secondary structure prediction, the mutated protein presents a slightly different structure when the amino acid residue changed from G to T. Figures 8 and 9 illustrate both the predicted models showing that the mutation would affect the tertiary structure of the protein. The comparison of residues 45-55 between MUT31136 and MOD30336 showed that this portion of the protein with the mutation stretches out with repercussions to the preceding helix. This result suggests that the mutation of the single amino acid from G to T, with consecutive stretching cycles on the 3D structure of the protein, tends to make the protein assume new functions.

### Prediction of Protein-Related Ontologies

The analysis of protein ontologies indicates different functions between the wild-type and mutated proteins, owing to their changed structures. As shown in Table 4, the wild-type variant of the protein is linked with a high probability (.978≤*P*≤1) to both catalytic and transferase activities. The mutated variant of the protein presents a remarkable change in its functionality trend: even if usually the scores below 0.5 are interpreted as negative predictions, in an evolutionary context, the decrease in probability of the transferase activity (from 0.98 to 0.375) to favor the binding function (from 0.004 to 0.132) is not regarded as negligible. The contextual inversion of tendency of transferase to binding activity suggests that the episodic evolutionary mutation aims to improve the binding ability of the protein.

**Table 4:** Classification report showing the predicted functions of both (mutated and wild-type) protein sequences and related scores. Only positive scores are reported.

Gene ontology terms and function		Score	
		Wild-type protein sequence	Mutated protein sequence
GO:0003674	Molecular function	1	1
GO:0003824	Catalytic function	1	0.998
GO:0016740^a^	Transferase activity	0.978	0.375
GO:0016829	Lyase activity	0.017	—^b^
GO:0022891	Transmembrane	0.07	—^b^
GO:0005488^a^	Binding activity	0.004	0.132
GO:0022892	Transmembrane transport activity	0.001	0.001

^a^Ontological functions subjected to inverted tendency.

^b^Unpredicted function.

## Discussion

### Principal Findings

SAR-CoV-2, the virus known to cause the COVID-19 pandemic, has many peculiar characteristics, such as rapidly accumulating mutations, compared to other coronaviruses [25]. Specifically, the prevalence of single nucleotide transitions as the major mutational type of SAR-CoV-2 across the world has been shown previously [7]. In this study, we conducted evolutionary analyses on the mutations to determine whether SARS-CoV-2 genomes from different countries in the same meridian might have specific variation patterns. We found that codon 9628 was under episodic selective pressure for all 4 countries in the same meridian. This would affect RNA secondary structure and, consequently, the protein product, with T (threonine) changing to G (glycine) in protein position 50. This position is located close to the predicted transmembrane region. Mutation analysis revealed that a change from G (glycine) to D (aspartic acid) may confer a new function to the protein, that is, binding activity, which in turn may be responsible for attaching the virus to human eukaryotic cells. These bioinformatics findings may help in better designing in vitro (wet lab) and in vivo (animal model) experiments to determine protein variants associated with the virulence of the virus. Therefore, these findings may eventually facilitate vaccine design and successful antiviral strategies. For example, the results of this study suggest the need for site-directed mutagenesis and animal experiments to validate the anticipated effects.

Mercatelli and Georgi [7] demonstrated that clade G, prevalent in Europe, carries a D614G mutation in the spike protein, which is responsible for the initial interaction of the virus with the host human cell. Other studies have also shown different mutation locations among strains isolated from different continents. Mutations at positions 2891, 3036, 14408, 23403, and 28881 are predominantly observed in European strains, whereas those located at positions 17746, 17857, and 18060 are exclusively present in North American strains of SARS-CoV-2 [26]. Their findings suggest that the virus is evolving and that European, North American, and Asian strains of the virus might coexist, with each characterized by different mutation patterns.

Furthermore, a comparison of viral genomes of SARS-CoV-2 strains from 13 countries identified differences in the protein-coding sequences. For example, an Indian strain showed a mutation in the spike glycoprotein at R408I and in the replicase polyprotein at I671T, P2144S, and A2798V, whereas the spike protein of Spain and South Korean strains carried an F797C and a S221W mutation, respectively [27]. Moreover, recently conducted integrative analyses of SARS-CoV-2 genomes of strains from different geographical locations reveal unique features that are potentially consequential to host-virus interaction and pathogenesis [28]. However, the most recent study of genomic diversity and hotspot mutations in 30,983 SARS-CoV-2 genomes indicates that unlike the influenza virus or HIV, SARS-CoV-2 has a low mutation rate, which makes the development of an effective global vaccine very likely [29]. The study determined several hotspot mutations across the whole SARS-CoV-2 genome. In all, 14 nonsynonymous hotspot mutations (whose prevalence of mutations is >10%) have been identified at different locations along the viral genome: 8 in ORF1ab polyprotein (in nsp2, nsp3, transmembrane domain, RdRp, helicase, exonuclease, and endoribonuclease), 3 in nucleocapsid protein, and 1 in each of the 3 proteins spike, ORF3a, and ORF8. Moreover, 36 nonsynonymous mutations were identified in the receptor-binding domain of the spike protein with a low prevalence (<1%) across all genomes [29].

### Conclusions

All these findings highlight the importance of studying the relationship of geographical locations of SARS-CoV-2 isolates and mutations in their genomes, because the relationship can also be confirmed by phylogenetic tree analyses for elucidation of lineages and clusters based on the geographic locations. In conclusion, this genome evolutionary analysis revealed that codon 9628 is under episodic selective pressure for SARS-CoV-2 strains isolated from all 4 countries (Italy, Sweden, Congo, and Brazil) of the same geographical meridian.

## Abbreviations

GISaid: Global Initiative on Sharing All Influenza Data
MEME: mixed effects model of evolution
MERS-CoV: Middle East respiratory syndrome coronavirus
ORF: open reading frames
